# Identification of significant genes as prognostic markers and potential tumor suppressors in lung adenocarcinoma via bioinformatical analysis

**DOI:** 10.1186/s12885-021-08308-3

**Published:** 2021-05-26

**Authors:** Mingze Lu, Xiaowen Fan, Weilin Liao, Yijiao Li, Lijie Ma, Mu Yuan, Rui Gu, Zhengdao Wei, Chao Wang, Hua Zhang

**Affiliations:** 1 Department of Human Resources, General Hospital of Western Theater Command, Chengdu, 610083 China; 2 Department of Thoracic Surgery, General Hospital of Western Theater Command, Chengdu, 610083 China; 3 Department of Anesthesiology, The People’s Hospital of Leshan, Leshan, 614000 China; 4 Department of Pulmonary and Critical Care Medicine, General Hospital of Western Theater Command, Chengdu, 610083 China; 5 Department of Scientific Research & Training, General Hospital of Western Theater Command, Chengdu, 610083 China; 6 Basic Medical Laboratory, General Hospital of Western Theater Command, Chengdu, 610083 China; 7 Department of Outpatient, General Hospital of Western Theater Command, Chengdu, 610083 China; 8 Department of Pathology, General Hospital of Western Theater Command, NO.270 Tianhui Road, Rongdu Avenue, Jinniu District, Chengdu, 610083 China; 9 State Key Laboratory of Biotherapy, Sichuan University, Chengdu, 610041 China

**Keywords:** Bioinformatics analysis, Prognostic markers, Tumor suppressors, Lung adenocarcinoma

## Abstract

**Background:**

Lung adenocarcinoma (LAC) is the predominant histologic subtype of lung cancer and has a complicated pathogenesis with high mortality. The purpose of this study was to identify differentially expressed genes (DEGs) with prognostic value and determine their underlying mechanisms.

**Methods:**

Gene expression data of GSE27262 and GSE118370 were acquired from the Gene Expression Omnibus database, enrolling 31 LAC and 31 normal tissues. Common DEGs between LAC and normal tissues were identified using the GEO2R tool and Venn diagram software. Next, the Database for Annotation, Visualization, and Integrated Discovery (DAVID) was used to analyze the Gene Ontology and Kyoto Encyclopedia of Gene and Genome (KEGG) pathways. Then, protein-protein interaction (PPI) network of DEGs was visualized by Cytoscape with Search Tool for the Retrieval of Interacting Genes and central genes were identified via Molecular Complex Detection. Furthermore, the expression and prognostic information of central genes were validated via Gene Expression Profiling Interactive Analysis (GEPIA) and Kaplan-Meier analysis, respectively. Finally, DAVID, real-time PCR and immunohistochemistry were applied to re-analyze the identified genes, which were also further validated in two additional datasets from ArrayExpress database.

**Results:**

First, 189 common DEGs were identified among the two datasets, including 162 downregulated and 27 upregulated genes. Next, Gene Ontology and KEGG pathway analysis of the DEGs were conducted through DAVID. Then, PPI network of DEGs was constructed and 17 downregulated central genes were identified. Furthermore, the 17 downregulated central genes were validated via GEPIA and datasets from ArrayExpress, and 12 of them showed a significantly better prognosis. Finally, six genes were identified significantly enriched in neuroactive ligand-receptor interactions (EDNRB, RXFP1, P2RY1, CALCRL) and Rap1 signaling pathway (TEK, P2RY1, ANGPT1) via DAVID, which were further validated to be weakly expressed in LAC tissues via RNA quantification and immunohistochemistry analysis.

**Conclusions:**

The low expression pattern and relation to prognosis indicated that the six genes were potential tumor suppressor genes in LAC. In conclusion, we identified six significantly downregulated DEGs as prognostic markers and potential tumor suppressor genes in LAC based on integrated bioinformatics methods, which could act as potential molecular markers and therapeutic targets for LAC patients.

**Supplementary Information:**

The online version contains supplementary material available at 10.1186/s12885-021-08308-3.

## Background

Lung cancer remains the leading cause of cancer-related deaths in men and women worldwide [1, 2]. In China, both incidence and mortality from lung cancer continue to increase, which poses a significant threat to public health [3]. The complicated pathogenesis of lung cancer result from a variety of risk factors, most commonly include lifestyle, environmental, occupational exposure, and genetic factors [1]. Adenocarcinoma is the predominant histologic subtype of lung cancer both in men and women [1, 4]. However, despite advances in tumor biology and treatment, the five-year overall survival rate is approximately 15.7% [5], and varies markedly depending on the stage when the diagnosis is made [6]. Thus, it is essential to identify specific molecular markers and develop a more personalized therapy for lung adenocarcinoma (LAC) to improve early prediction and outcomes.

To date, molecular markers have been widely studied for the detection and prognosis of LAC. Runt-related transcription factor 3 (RUNX3) [7], estrogen receptor [8], and chemokine receptor [9, 10] have been identified as good prognostic markers, whose high expression is significantly correlated with an increase in disease-free survival in LAC patients. In addition, Ets-1 [11], Kruppel-like factor 6 (KLF6) [12], eukaryotic initiation factor 4E (eIF4E) [13], Nectin-like molecule-5 (Necl-5) [14], and histone deacetylases (HDACs) [15, 16] have been demonstrated as poor prognostic markers [17]. Furthermore, molecular markers have been tested as targets of specific therapies for LAC patients. Epidermal growth factor receptor (EGFR) insertions and deletions have been found in approximately 15% of LAC patients in the United States [18], which indicates a favorable sensitivity to tyrosine kinase inhibitors towards EGFR [19]. KRAS mutations have been commonly found in smokers and appear to confer a worse prognosis [18]. Drugs that target KRAS mutations are actively testing in clinical trials [19]. Additional gene mutations, such as BRAF mutations, HER2 mutations, ROS1 translocations, and ALK gene rearrangements, could also be targets in LAC patients.

However, an increase in molecular markers for lung adenocarcinoma is still in urgent demand. The datasets of gene expression profiles in the Gene Expression Omnibus (GEO) are far from being excavated and contain a great deal of information regarding LAC. The bioinformatic analysis provides a powerful and comprehensive tool for analyzing gene expression data from multiple datasets. Thus, in this study, we first searched the gene expression profiling datasets of LAC in GEO and finally chose GSE27262 and GSE118370 for bioinformatic analysis. Second, we applied the GEO2R and Venn diagram software to identify the common differentially expressed genes (DEGs) between the two datasets. Then, Gene Ontology and pathway enrichment were analyzed through the Database for Annotation, Visualization and Integrated Discovery (DAVID), including the molecular function (MF), cellular component (CC), biological process (BP), and Kyoto Encyclopedia of Gene and Genome (KEGG) pathways [20]. Furthermore, we constructed a protein-protein interaction (PPI) network and then applied the Cytoscape Molecular Complex Detection (MCODE) to identify the core genes in the PPI network. Moreover, we validated the core gene’s expression between LAC tissues and normal lung tissues via Gene Expression Profiling Interactive Analysis (GEPIA) and ArrayExpress datasets. In addition, these core genes were further analyzed for significant prognostic information based on the Kaplan-Meier online database. Thus, 12 core genes were qualified and KEGG pathway enrichment was re-analyzed. Finally, six genes were generated, which were mainly enriched in neuroactive ligand-receptor interactions and Rap1 signaling pathway, and their further expressions were validated via RNA quantification and immunohistochemistry analysis in tissue samples. The low expression of the six genes and their relation to prognosis in LAC indicated that they were potential tumor suppressor genes. In conclusion, our bioinformatics study identified useful and potential tumor suppressor genes that could potentially act as biomarkers and effective targets for LAC patients.

## Methods

### Microarray data information

NCBI-GEO is a widely used public database and provides gene expression profile of numerous cancers for study. The keywords in the search process were as follows: lung cancer, non-small cell lung cancer, lung adenocarcinoma, and GPL570. The following criteria were used to screen the datasets and ensure relevant data were recorded: (I) the sample includes lung adenocarcinoma and paired adjacent tissues; (II) the study type is expression profiling by array; (III) the species is limited to *Homo sapiens*; (IV) access to raw data is allowed. We obtained the gene expression data of GSE27262 and GSE118370 in lung adenocarcinoma and paired normal lung tissues for bioinformatics analysis. Microarray data of GSE27262 and GSE118370 were based on GPL570 Platforms ([HG-U133_Plus_2] Affymetrix Human Genome U133 Plus 2.0 Array), including 25 LAC tissues and 25 paired normal lung tissues, 6 LAC tissues and 6 paired normal lung tissues, respectively. Another two datasets from ArrayExpress database, E-GEOD-30219 and E-GEOD-19188, which include specimens from LAC of variable TNM stages, were enrolled to further validate the expression of identified genes.

### Data processing of DEGs

Robust multi-array average (RMA) and MicroArray Suite (MAS) approach was performed for background correction and normalization. The GEO2R online tools [21] on the NCBI-GEO website, which using the GEO query and limma R packages to analyze high-throughput genomic data, were used to identify DEGs between the LAC specimen and normal lung specimen with |log_2_FC| > 2 and an adjusted *P* value < 0.05 [20]. Then, the raw data were analyzed using Venn software online to identify the common DEGs among the original two datasets. DEGs with log_2_FC < 0 were considered as downregulated genes, while the DEGs with log_2_FC > 0 were considered as upregulated genes [20].

### Gene ontology and KEGG pathway enrichment analysis

DAVID [22] is an online bioinformatics tool that integrates the function of Gene Ontology and KEGG pathway enrichment analysis [23, 24]. Through DAVID, we identified the unique biological properties of the common DEGs and visualized the DEGs enrichment of molecular function (MF), cellular components (CC), biological processes (BP), and KEGG pathways (*P* < 0.05).

### PPI network construction and module analysis

The Search Tool for the Retrieval of Interacting Genes (STRING) online tool [25] was used to evaluate PPI information. Thereafter, Cytoscape [26] was used to visualize the potential correlation and interaction between these DEGs (maximum number of interactors = 0 and confidence score ≥ 0.4). In addition, the MCODE app was applied to inspect the central modules of the PPI network (degree cutoff = 2, max. Depth = 100, k-core = 2, and node score cutoff = 0.2) [20]. PPI network properties, such as node degree and betweenness centrality, were visualized by shape size and label font size, respectively.

### Survival and RNA sequencing analysis

The Kaplan-Meier plotter is a useful website tool, which contains considerable information on several cancers, including breast and lung cancer [27]. Survival analysis was conducted using the Kaplan-Meier plotter, and the log-rank *P* value and hazard ratio (HR) with 95% confidence intervals were computed and shown on the plot [20]. To further validate these DEGs with significant survival outcomes, we applied the Gene Expression Profiling Interactive Analysis (GEPIA) website to analyze the RNA sequencing data based on the GTEx projects and TCGA database [28].

### RNA quantification

Total RNA was extracted with Trizol reagent (Invitrogen) and reverse-transcribed using the PrimeScript™ RT reagent kit (Takara). Quantitative real-time PCR analysis was performed on a LightCycler (Roche) with the TB Green® Premix Ex Taq™ II (Takara). Data were normalized to *GAPDH* expression. The primers used for real-time PCR were as follows: *GAPDH* (forward: 5′-GGA GCG AGA TCC CTC CAA AAT-3′, reverse: 5′-GGC TGT TGT CAT ACT TCT CAT GG-3′), *EDNRB* (forward: 5′-CTG GCC ATT TGG AGC TGA GA-3′, reverse: 5′-CAG AAC CAC AGA GAC CAC CC-3′), *RXFP1* (forward: 5′-GGA CCT GAA GGA GCT GTC AC-3′, reverse: 5′-AGG CTG AGA GAC TTG AGT TTG A-3′), *P2RY1* (forward: 5′-CCG TCT CCT CGT CGT TCA AA-3′, reverse: 5′-ACG TAC AAG AAG TCG GCC AG-3′), *CALCRL* (forward: 5′-CCC ACC TTG CTT GTG GGT AA-3′, reverse: 5′-GTC AAG ACC CAG TCC AGC TC-3′), *TEK* (forward: 5′-CCA GCC CTG CTG ATA CCA AA-3′, reverse: 5′-AGG CAA GAA GGA ACA GCA CA-3′), and *ANGPT1* (forward: 5′-TCC AGG AGC TGG AAA AGC AA-3′, reverse: 5′-TGC AAA GAT TGA CAA GGT TGT GG-3′).

### Immunohistochemical (IHC) staining

IHC staining was applied to detect the protein level of certain genes and performed according to standard protocols using the following antibodies: anti-EDNRB (bs-2363R, 1:500), anti-RXFP1 (bs-15368R, 1:500), anti-P2RY1 (EM1710–48, 1:200), anti-CALCRL (BA1572–1, 1:200), anti-TEK (bs-1300R, 1:500), and anti-ANGPT1 (bs-0800R, 1:500).

## Results

### Identification of DEGs in lung adenocarcinoma

**Fig. 1:**
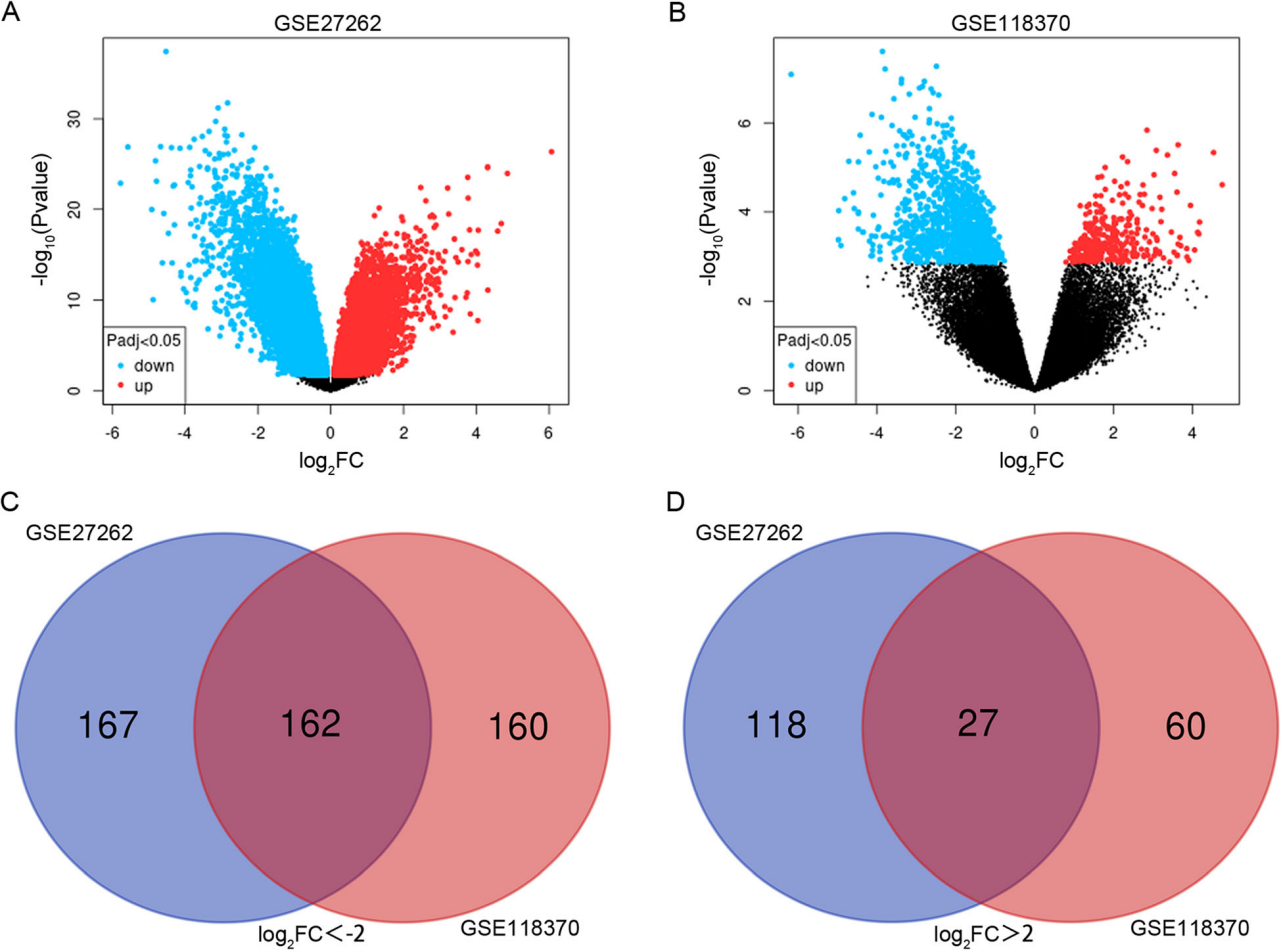
Identification of 189 common DEGs among GSE27262 and GSE118370 datasets by Venn diagram software. Different colors represent different datasets. **a**, **c** 162 DEGs were downregulated among the two datasets (log_2_FC < −2). **b**, **d** 27 DEGs were upregulated among the two datasets (log_2_FC > 2)

In the present study, the gene expression data of GSE27262 and GSE118370 were chosen for bioinformatic analysis, including 31 LAC tissues and 31 normal lung tissues. Using the GEO2R online tool, we obtained 474 and 409 DEGs from GSE27262 and GSE118370 (Fig. 1a & b), respectively (|log_2_FC| > 2 and adjusted *P* value < 0.05). Then, we applied Venn diagram software to identify the common DEGs among the two datasets. Results showed that 189 common DEGs were identified, including 162 downregulated genes (log_2_FC < 0) and 27 upregulated genes (log_2_FC > 0) in LAC tissues (Table 1, S1 & S2, and Fig. 1c & d).

**Table 1:** All 189 common differentially expressed genes (DEGs) were identified from two profile datasets, including 162 downregulated genes and 27 upregulated genes in lung adenocarcinoma tissues compared to normal lung tissues

DEGs	Gene Name
Downregulated	HBA2///HBA1 ACE SCN7A FMO2 RTKN2 EMCN SLC19A3 HSPB6 SOX7 MYZAP ADARB1 AOC3 CCM2L SFTPC PPP1R14A FRMD3 P2RY1 WISP2 MFAP4 KCNT2 SVEP1 TTN SEMA3G ERG SLC6A4 PECAM1 TCF21 TGFBR3 KCNK3 HHIP ADH1B ARHGEF26 SH3GL3 ARHGAP6 GIMAP8 SCARA5 NFASC LINC00968 ASPA BTNL9 PCAT19 NDRG4 FABP4 IGSF10 EDNRB ACVRL1 GPC3 FCN3 CDO1 MYCT1 LIN7A KANK3 SDPR TEK GRIA1 STX11 LINC00312 GRK5 FAM107A CCDC85A DACH1 VGLL3 EML1 CCBE1 GPX3 AGER GUCY1A2 GHR PALM2-AKAP2///AKAP2 ANXA8L1///ANXA8 SOX17 STXBP6 RGCC VWF PDE8B S1PR1 SEMA5A GIMAP1 CD300LG ABI3BP CD93 TIE1 EMP2 LYVE1 KIAA1462 VIPR1 LEPROT///LEPR SLC5A9 SH2D3C CYYR1 STARD13 EPAS1 RAMP3 CLIC5 SPOCK2 KL AKAP12 CD36 SLIT2 TRHDE FGFR4 FHL5 PDE5A ADGRL3 MME LDB2 SHANK3 ROBO4 SPTBN1 CALCRL SLC14A1 TMTC1 CAV1 CLEC1A RXFP1 SMAD9 RASIP1 SGCG PPBP JAM2 TBX3 PTPRB AFAP1L1 QKI FOXF1 ACADL SEMA6D CLDN18 ADGRL2 ANKRD29 FENDRR AQP4 PIR-FIGF///FIGF CDH5 HEG1 PDK4 GPM6A ITGA8 COL6A6 TNNC1 IL1RL1 ECSCR ANGPT1 ADRA1A SMAD6 DPP6 FAT3 SEMA6A MCEMP1 HBB LOC100996760 NTNG1 GNG11 FHL1 RHOJ TMEM100 SSTR1 DUOX1 KLF4 TRDV3 GNLY KLRF1
Upregulated	KIF26B SPINK1 SGPP2 GDF15 MMP7 C1orf53 TMEM45B TOX3 GJB2 AFAP1-AS1 PROM2 CD24 ABCC3 TMPRSS4 HS6ST2 CP SERPIND1 BLACAT1 MMP1 NEK2 CLIC6 HABP2 WFDC2 SIX1 TFAP2A AGR2 COL10A1

### Gene ontology and KEGG analysis of DEGs in lung adenocarcinoma

To examine the biological properties of the 189 DEGs, Gene Ontology and KEGG analysis were conducted via DAVID software. Results of Gene Ontology analysis indicated that 1) for biological processes (BP), downregulated DEGs were significantly enriched in angiogenesis, vasculogenesis signal transduction, receptor internalization, and neural crest cell migration, and upregulated DEGs were enriched in collagen catabolic processes, proteolysis, sensory perception of sound, and embryonic cranial skeleton morphogenesis; 2) for cell components (CC), downregulated DEGs were significantly enriched in the integral components of membrane, plasma membrane, proteinaceous extracellular matrix and cell-cell junctions, and upregulated DEGs were significantly enriched in extracellular space, extracellular regions, extracellular exosomes, and the proteinaceous extracellular matrix; 3) for molecular function (MF), downregulated DEGs were enriched in semaphoring receptor binding, chemorepellent activity, heparin-binding, and calcium ion-binding, and upregulated DEGs were enriched in endopeptidase inhibitor activity, serine-type endopeptidase inhibitor activity (*P* < 0.05, Table 2).

**Table 2:** Gene Ontology analysis of differentially expressed genes in lung adenocarcinoma

Expression	Category	Term	Count	%	*p*-Value	FDR
Downregulated	GOTERM_BP_DIRECT	GO:0001525 ~ angiogenesis	10	6.2	3.06E-07	4.71E-04
	GOTERM_BP_DIRECT	GO:0001570 ~ vasculogenesis	7	4.3	3.34E-06	0.005135
	GOTERM_BP_DIRECT	GO:0007165 ~ signal transduction	12	7.4	1.26E-04	0.193754
	GOTERM_BP_DIRECT	GO:0031623 ~ receptor internalization	5	3.1	1.45E-04	0.222577
	GOTERM_BP_DIRECT	GO:0001755 ~ neural crest cell migration	5	3.1	3.69E-04	0.566138
	GOTERM_CC_DIRECT	GO:0016021 ~ integral component of membrane	49	30.3	1.48E-04	0.16707
	GOTERM_CC_DIRECT	GO:0005886 ~ plasma membrane	25	15.4	8.82E-04	0.993318
	GOTERM_CC_DIRECT	GO:0005578 ~ proteinaceous extracellular matrix	6	3.7	0.008111	8.80424
	GOTERM_CC_DIRECT	GO:0005911 ~ cell-cell junction	4	2.5	0.047922	42.63677
	GOTERM_MF_DIRECT	GO:0030215 ~ semaphorin receptor binding	4	2.5	2.31E-04	0.280668
	GOTERM_MF_DIRECT	GO:0045499 ~ chemorepellent activity	4	2.5	8.56E-04	1.037711
	GOTERM_MF_DIRECT	GO:0008201 ~ heparin binding	4	2.5	0.040365	39.47803
Upregulated	GOTERM_BP_DIRECT	GO:0030574 ~ collagen catabolic process	3	11.1	0.003145	3.886365
	GOTERM_BP_DIRECT	GO:0006508 ~ proteolysis	5	18.5	0.003709	4.568267
	GOTERM_BP_DIRECT	GO:0007605 ~ sensory perception of sound	3	11.1	0.012967	15.14675
	GOTERM_BP_DIRECT	GO:0048701 ~ embryonic cranial skeleton morphogenesis	2	7.4	0.039861	40.06517
	GOTERM_CC_DIRECT	GO:0005615 ~ extracellular space	8	29.6	0.001014	0.992618
	GOTERM_CC_DIRECT	GO:0005576 ~ extracellular region	8	29.6	0.002863	2.780239
	GOTERM_CC_DIRECT	GO:0070062 ~ extracellular exosome	9	33.3	0.017782	16.17818
	GOTERM_CC_DIRECT	GO:0005578 ~ proteinaceous extracellular matrix	3	11.1	0.044503	36.09452
	GOTERM_MF_DIRECT	GO:0004866 ~ endopeptidase inhibitor activity	3	11.1	0.001227	1.243792
	GOTERM_MF_DIRECT	GO:0004252 ~ serine-type endopeptidase activity	4	14.8	0.004242	4.240205
	GOTERM_MF_DIRECT	GO:0004867 ~ serine-type endopeptidase inhibitor activity	3	11.1	0.007004	6.913304

The results of KEGG analysis showed that downregulated DEGs were particularly enriched in neuroactive ligand-receptor interactions, cell adhesion molecules, axon guidance, hypertrophic cardiomyopathy, and vascular smooth muscle contractions, while upregulated DEGs were not enriched in any significant signaling pathways (*P* < 0.05, Table 3).

**Table 3:** KEGG pathway analysis of differentially expressed genes (downregulated) in lung adenocarcinoma

Pathway ID	Name	Count	%	*p*-Value	Genes
ptr04080	Neuroactive ligand-receptor interaction	10	6.2	0.001298	EDNRB, S1PR1, RXFP1, GRIA1, SSTR1, P2RY1, ADRA1A, CALCRL, VIPR1, GHR
ptr04514	Cell adhesion molecules (CAMs)	7	4.3	0.003249	CLDN18, ITGA8, PECAM1, NTNG1, NFASC, JAM2, CDH5
ptr04360	Axon guidance	6	3.7	0.008075	SEMA5A, SEMA6A, SEMA6D, SEMA3G, NTNG1, SLIT2
ptr05410	Hypertrophic cardiomyopathy (HCM)	5	3.1	0.009096	ACE, SGCG, TNNC1, ITGA8, TTN
ptr04270	Vascular smooth muscle contraction	5	3.1	0.033494	RAMP3, GUCY1A2, ADRA1A, CALCRL, PPP1R14A

### The PPI network construction and modular analysis of DEGs

**Fig. 2:**
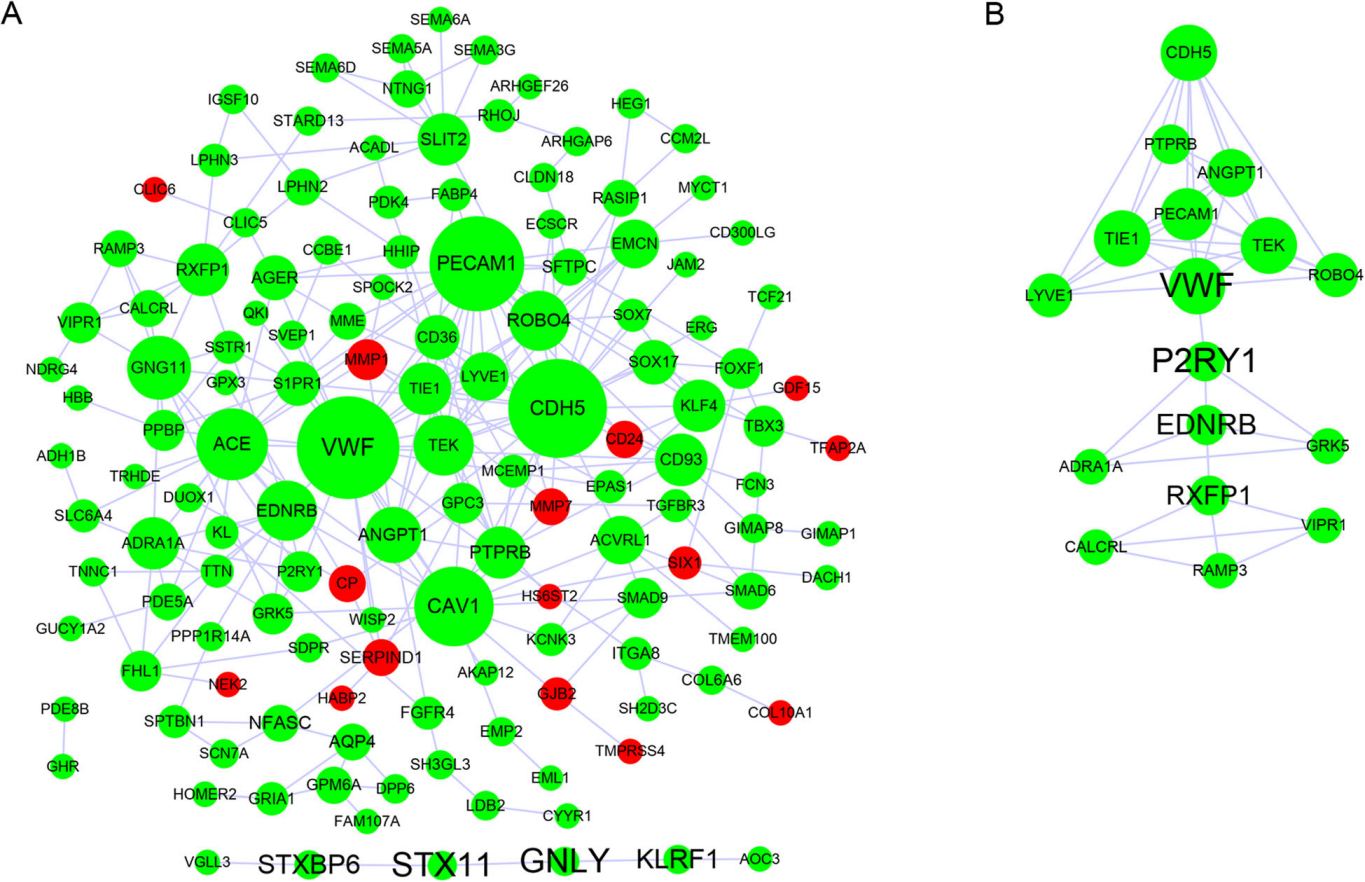
Common DEGs PPI network was constructed using the STRING online database and Cytoscape software. **a** There were 137 nodes and 254 edges in the PPI network. The nodes represent proteins; the edges represent the interaction between proteins; green circles represent downregulated DEGs, and red circles represent upregulated DEGs. **b** Modular analysis via MCODE (degree cutoff = 2, max. Depth = 100, k-core = 2, and node score cutoff = 0.2). In total, 17 central nodes were screened. Circle size represents node degree, and label font size represents betweenness centrality

To analyze the PPI information of the 189 DEGs, STRING online database and Cytoscape software were used to construct the PPI network complex. In total, 137 DEGs were enrolled in the PPI network, which included 137 nodes and 254 edges, including 122 downregulated and 15 upregulated genes (Fig. 2a and Table S3). There were 52 DEGs not presented in the DEGs PPI network. We then used Cytoscape MCODE to further screen the core genes, and results revealed that 17 central nodes, all of which were downregulated, were identified (Fig. 2b and Table S3).

### Analysis of 17 core genes via the GEPIA and Kaplan-Meier plotter

**Fig. 3:**
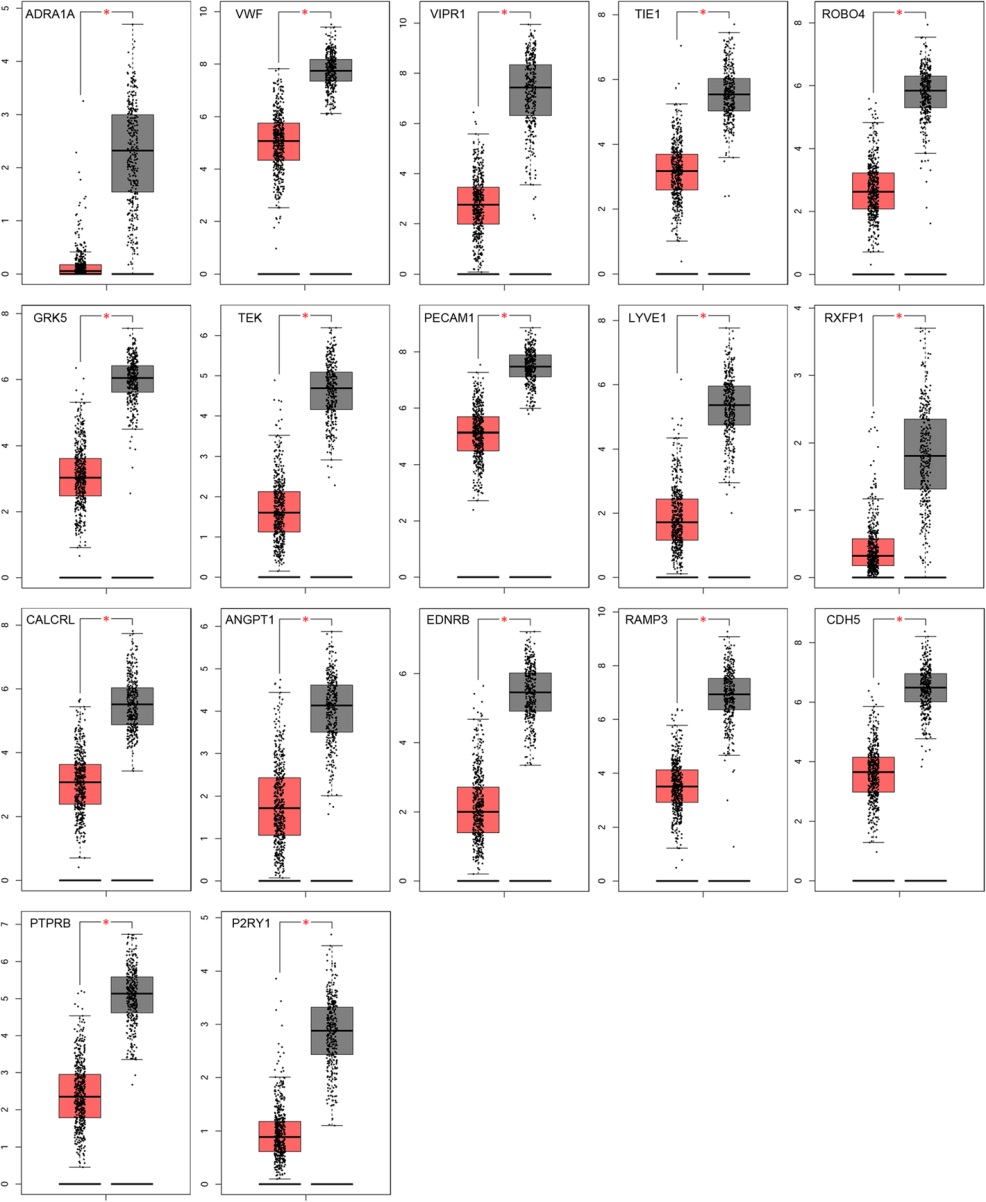
Expression levels of the 17 central genes in lung adenocarcinoma patients compared to healthy people. The GEPIA website was applied to validate the expression level of the 17 central genes between LAC patients and normal people. All 17 genes were lowly expressed in LAC specimens compared to normal specimens (*P < 0.05). Red indicates LAC tissues (*n* = 483) and gray indicates normal tissues (*n* = 347)

**Fig. 4:**
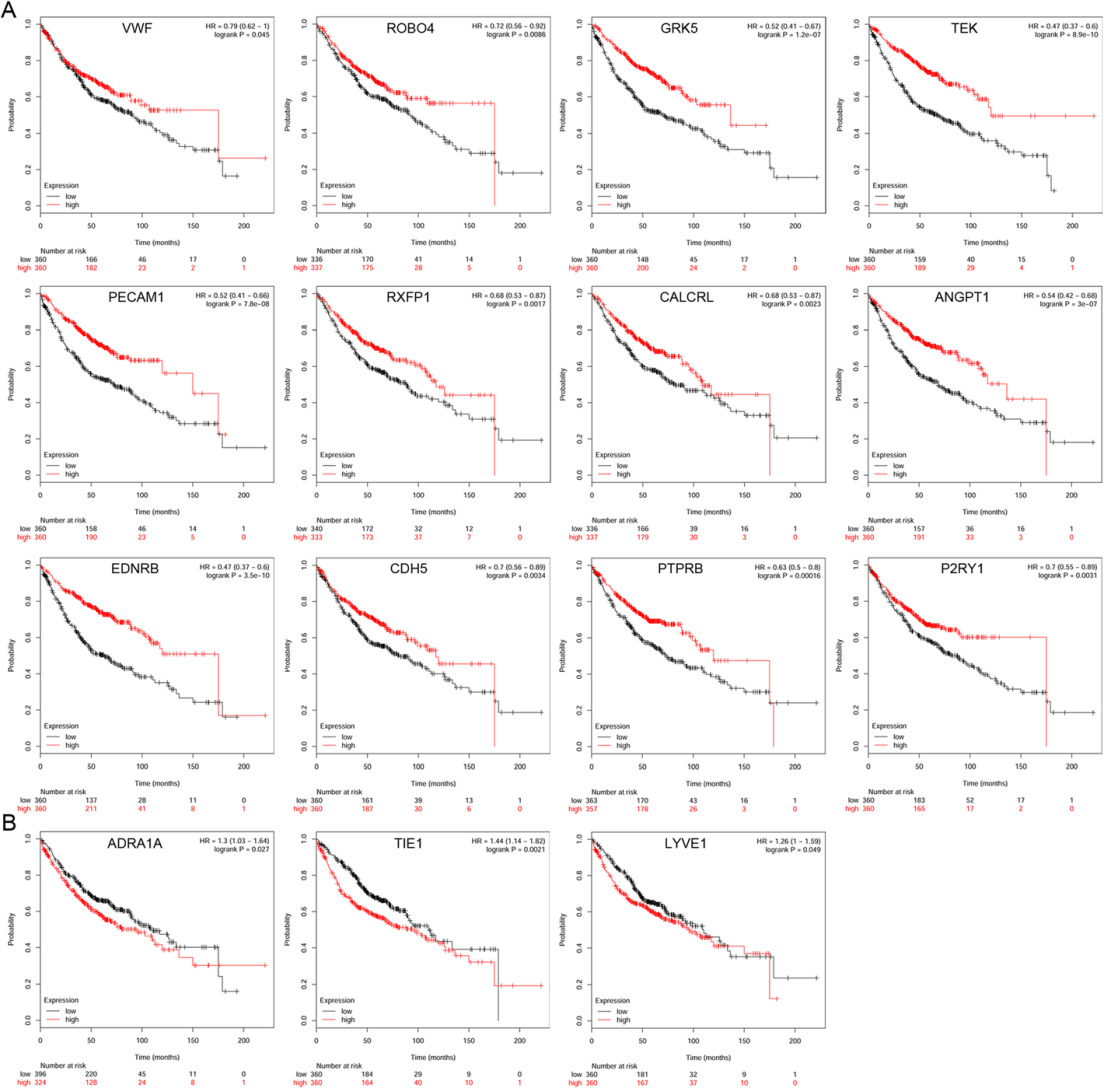
Prognostic information of the 17 central genes in lung adenocarcinoma. The Kaplan-Meier plotter online tools were used to analyze the prognostic information of the 17 central genes. **a** High expression of 12 of the 17 genes had a significantly better survival rate (*P* < 0.05). **b** High expression of ADRA1A, TIE1, and LYVE1 showed a significantly worse survival rate (*P* < 0.05)

To further validate the significance of the 17 central genes, GEPIA and the Kaplan-Meier plotter were utilized to identify the expression data and survival data, respectively. GEPIA expression data showed that all 17 genes were lowly expressed in LAC tissues compared to normal lung tissues (*P* < 0.05, Table 4 and Fig. 3). The Kaplan-Meier plotter survival data showed that a high expression of 12 of the 17 genes resulted in a significantly better survival probability, while high expression of ADRA1A, TIE1, and LYVE1 had a significantly worse survival probability; VIPR1 and RAMP3 were not significantly different (*P* < 0.05, Table 5 and Fig. 4).

**Table 4:** Validation of the 17 central genes using GEPIA

Category	Genes
Genes with low expression in LAC (*P*<0.05)	ADRA1A VWF VIPR1 TIE1 ROBO4 GRK5 TEK PECAM1 LYVE1 RXFP1 CALCRL ANGPT1 EDNRB RAMP3 CDH5 PTPRB P2RY1

**Table 5:** Prognostic information of the 17 key candidate genes

Category	Genes
Genes with significantly better survival (*P* <0.05)	VWF ROBO4 GRK5 TEK PECAM1 RXFP1 CALCRL ANGPT1 EDNRB CDH5 PTPRB P2RY1
Genes with significantly worse survival (*P* <0.05)	ADRA1A TIE1 LYVE1
Genes without significant survival (*P* >0.05)	VIPR1 RAMP3

### Re-analysis of 12 core genes by KEGG, RNA quantification and immunohistochemistry

**Fig. 5:**
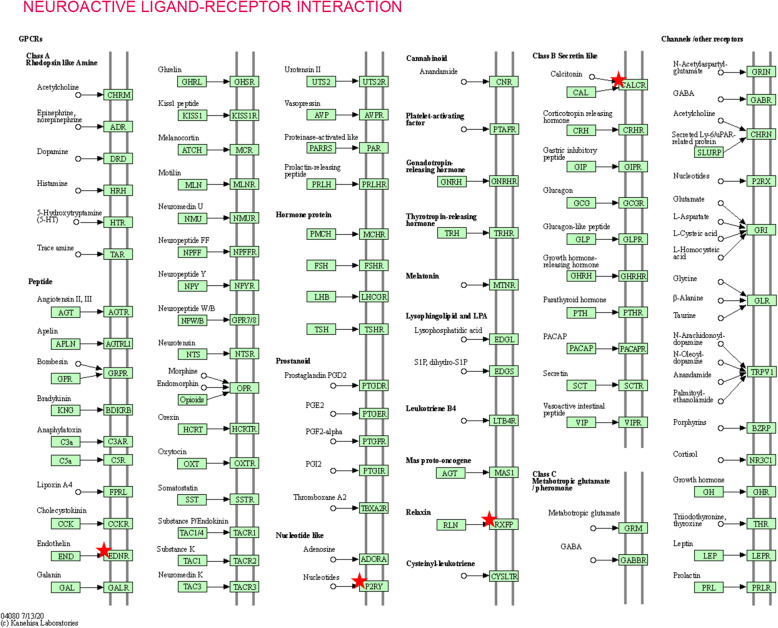
General information of neuroactive ligand-receptor interactions pathway. DAVID was used to re-analyze the 12 core DEGs for KEGG pathway enrichment. Four genes (EDNRB, RXFP1, P2RY1, and CALCRL) were enriched in neuroactive ligand-receptor interactions (*P* < 0.05)

**Fig. 6:**
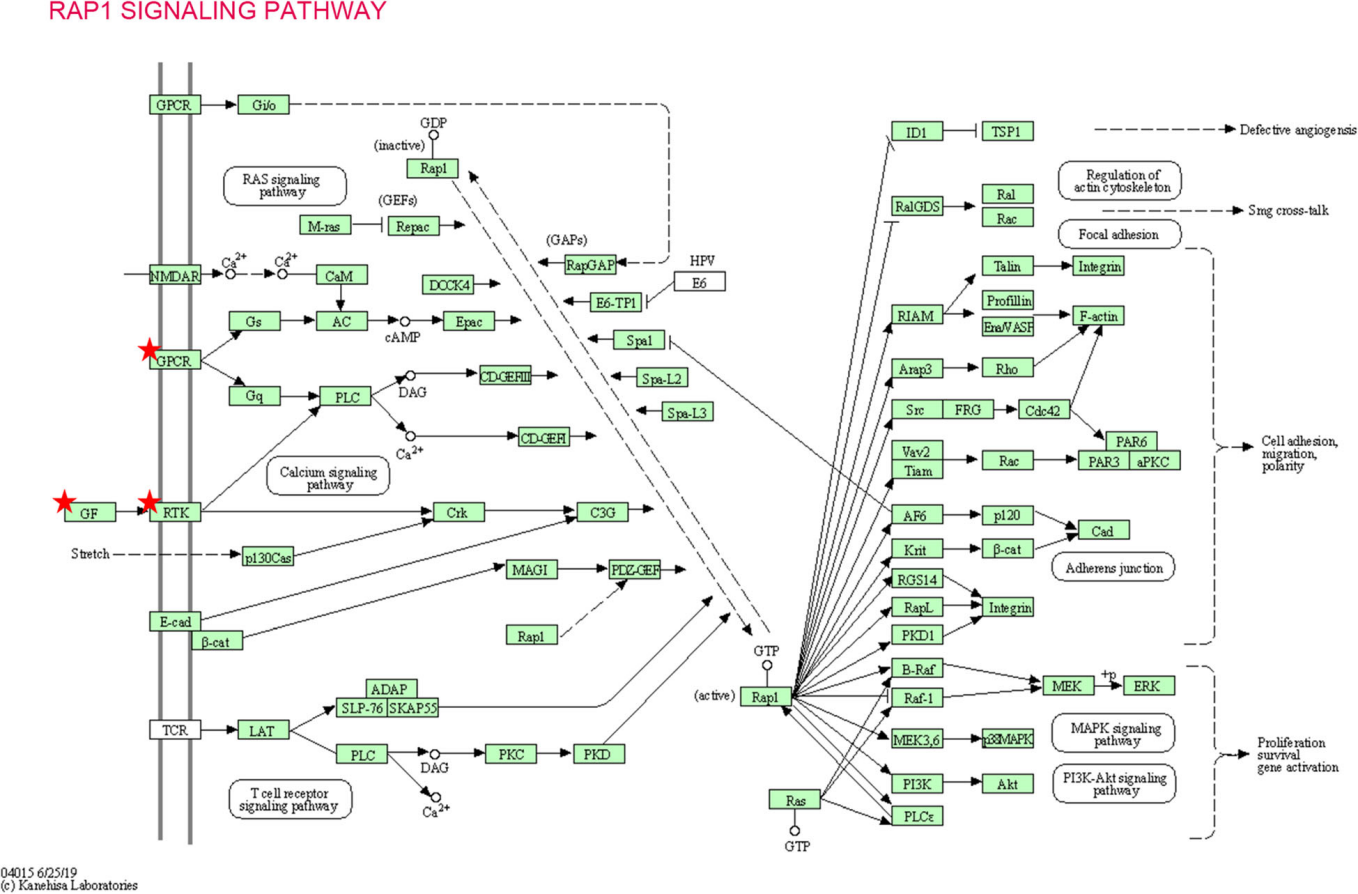
General information of Rap1 signaling pathway. DAVID was used to re-analyze the 12 core DEGs for KEGG pathway enrichment. Three genes (TEK, P2RY1, and ANGPT1) were enriched in Rap1 signaling pathway (*P* < 0.05)

To figure out the possible pathway of the 12 core DEGs, DAVID was used for KEGG pathway enrichment analysis. The results showed that six core genes were markedly enriched, in which four genes (EDNRB, RXFP1, P2RY1, and CALCRL) were enriched in neuroactive ligand-receptor interactions, and three genes (TEK, P2RY1, and ANGPT1) were enriched in the Rap1 signaling pathway (*P* < 0.05, Table 6 and Figs. 5 & 6).

**Table 6:** Re-analysis of the 12 selected genes via KEGG pathway enrichment

Pathway ID	Name	Count	%	*p*-Value	Genes
cfa04080	Neuroactive ligand-receptor interaction	4	33.3	0.005724	EDNRB RXFP1 P2RY1 CALCRL
cfa04015	Rap1 signaling pathway	3	25.0	0.035813	TEK P2RY1 ANGPT1

**Fig. 7:**
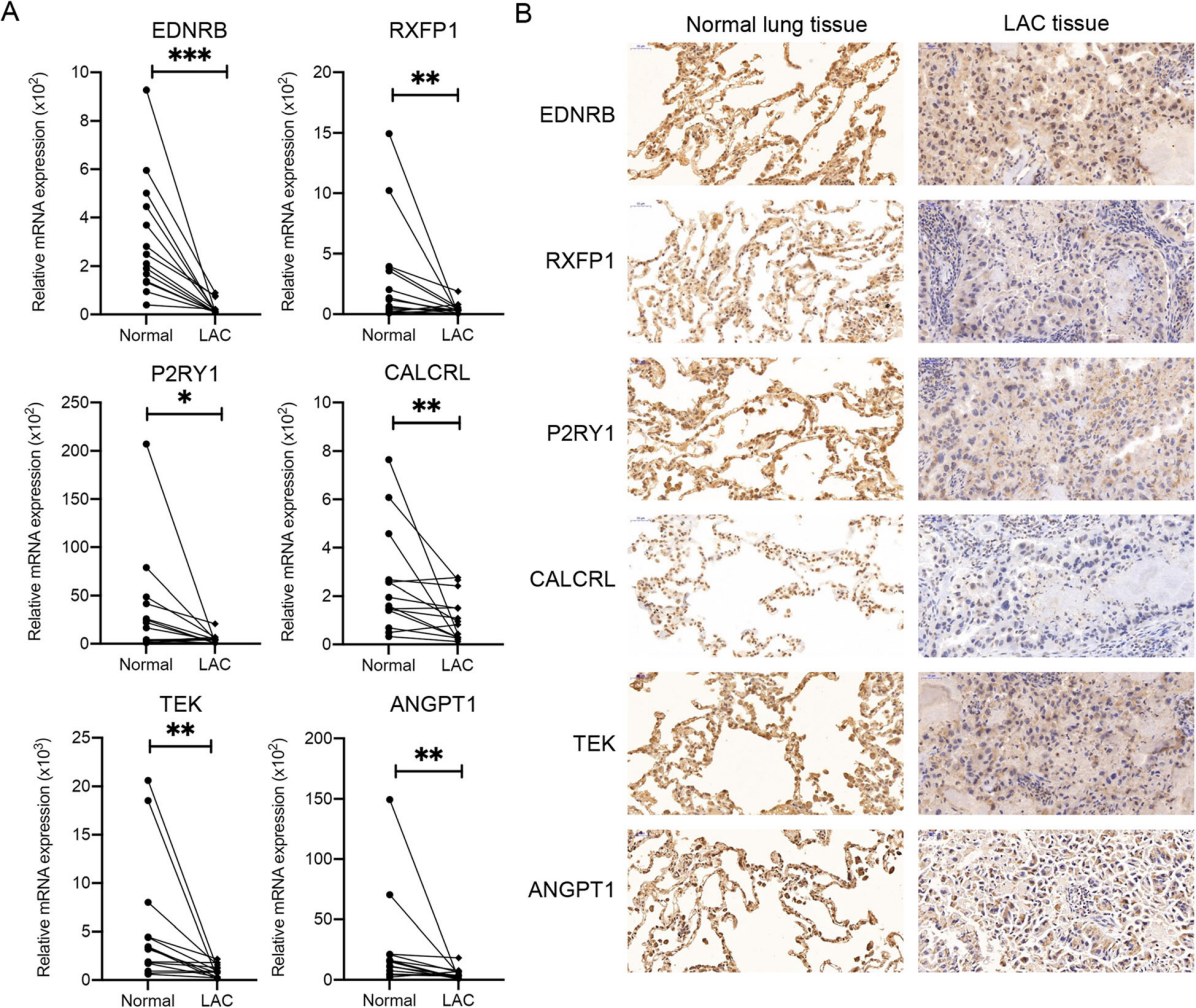
Validation of expression levels of EDNRB, RXFP1, P2RY1, CALCRL, TEK, and ANGPT1 in LAC patients. To further validate the expression level in LAC patients, six genes were re-analyzed via real-time PCR (**a**) and immunohistochemistry (**b**) analysis. Representative images of IHC staining were shown. Scale bar, 50 μm. Real-time PCR data were presented as mean ± SEM and the differences were estimated by Wilcoxon paired signed-rank test (*P < 0.05, **P < 0.01, ***P < 0.001). Data were normalized to *GAPDH* expression. All six genes were markedly weakly expressed in LAC tissue compared to adjacent normal tissue

Furthermore, we detected the expression levels of the above six genes in LAC specimens and normal lung specimens by RNA quantification and immunohistochemistry analysis. Results showed that all six genes were lowly expressed in LAC tissues compared to adjacent normal tissues (Fig. 7).

Finally, we further validated the expression of identified common DEGs, especially the 17 central genes in E-GEOD-30219 and E-GEOD-19188 datasets. The validated datasets were acquired from ArrayExpress database and included specimens from lung adenocarcinoma of variable TNM stage. Results showed that the vast majority of the previously identified 189 common DEGs and all 17 central genes were included in the DEGs of the two datasets with same expression pattern (Table S4 & S5).

## Discussion

In this study, we applied bioinformatics methods based on two gene expression profile datasets to identify additional useful prognostic molecular markers in lung adenocarcinoma. Thirty-one LAC specimens and thirty-one paired normal lung specimens were enrolled. Using GEO2R online tool and Venn software, we revealed 189 common DEGs (|log_2_FC| > 2 and adjusted *P* value < 0.05), including 162 downregulated and 27 upregulated DEGs. Gene Ontology and KEGG pathway enrichment analysis was conducted via DAVID. Gene Ontology analysis revealed that 1) for biological processes, downregulated DEGs were particularly enriched in angiogenesis, vasculogenesis signal transduction, receptor internalization, and neural crest cell migration, and upregulated DEGs were enriched in collagen catabolic process, proteolysis, sensory perception of sound, and embryonic cranial skeleton morphogenesis; 2) for cell components, downregulated DEGs were significantly enriched in integral components of the membrane, plasma membrane, proteinaceous extracellular matrix and cell-cell junctions, and upregulated DEGs were significantly enriched in the extracellular space, extracellular region, extracellular exosome, and proteinaceous extracellular matrix; 3) for molecular function, downregulated DEGs were significantly enriched in semaphoring receptor binding, chemorepellent activity, heparin-binding, and calcium ion-binding, and upregulated DEGs were enriched in endopeptidase inhibitor activity and serine-type endopeptidase inhibitor activity (*P* < 0.05). For pathway analysis, downregulated DEGs were particularly enriched in neuroactive ligand-receptor interactions, cell adhesion molecules, axon guidance, hypertrophic cardiomyopathy, and vascular smooth muscle contractions, while upregulated DEGs were not enriched in any significant signaling pathways (*P* < 0.05). Next, the PPI network complex of 137 nodes and 254 edges was constructed using the STRING and Cytoscape software. Thereafter, 17 central downregulated DEGs were screened from the PPI network by MCODE analysis. In addition, GEPIA analysis showed that all 17 genes were lowly expressed in LAC tissues (*P* < 0.05). Furthermore, through Kaplan-Meier analysis, we found that a high expression of 12 of the 17 genes displayed increased survival. Finally, we re-analyzed the 12 core genes via DAVID, RNA quantification, and immunohistochemistry. Six genes were markedly enriched and downregulated in LAC samples, in which four genes (EDNRB, RXFP1, P2RY1, and CALCRL) were enriched in neuroactive ligand-receptor interactions, and three genes (TEK, P2RY1, and ANGPT1) were enriched in the Rap1 signaling pathway (*P* < 0.05). Altogether, we identified six significant genes with good prognosis and tumor suppressor function in lung adenocarcinoma via bioinformatics analysis, which could be new molecular markers and effective targets for further research.

There was some evidence that EDNRB, RXFP1, ANGPT1, and TEK are closely related to lung diseases and cancer. For example, promoter hypermethylation of the EDNRB gene was found in several human tumors, including lung cancer. The endothelin receptor type B (EDNRB) gene encodes a G-protein coupled receptor and is regulated by the methylation of its CpG sites [29, 30]. Aberrant methylation of the EDNRB gene was detected in 32.9% (26 of 79) of lung cancer patients, which then decreased EDNRB expression and contributed to tumor progression [31]. These findings show that aberrant methylation of the EDNRB gene is highly prevalent in lung cancer. A previous study also identified EDNRB as a potential molecular marker for LAC via integrated bioinformatic analysis [17]. Recently, it was reported that EDNRB expression was significantly increased in chronic obstructive pulmonary disease (COPD) patients and was effectively reduced after celastrol treatment, which supposes an inflammation-related role of EDNRB in COPD [32].

Relaxin family peptide receptor-1 (RXFP1), also known as LGR7, is a leucine-rich repeat that contains a G-protein coupled receptor and is expressed in human and mouse lungs [33]. Evidence shows that RXFP1 appears to have a significant impact on lung diseases. A previous study revealed that Rxfp1-deficient mice had increased lung collagen accumulation as early as 1 month of age [34], indicating that RXFP1 could delay the age-related progression of pulmonary fibrosis. Further studies have demonstrated that RXFP1 protects against airway fibrosis during homeostasis but not against inflammation-induced fibrosis associated with chronic allergic airways [35]. More importantly, RXFP1 expression was reported to be directly associated with pulmonary function in patients with idiopathic pulmonary fibrosis (IPF), and results showed that patients with IPF and high RXFP1 expression are more sensitive to relaxin-based therapies [36].

Angiopoietin-1 (ANGPT1), a secreted glycoprotein, is a physiological angiogenesis promoter during embryonic development and has an enigmatic role in tumor angiogenesis [37]. The TEK receptor tyrosine kinase (TEK), also known as TIE2, is a receptor for ANGPT1 and belongs to the protein tyrosine kinase Tie2 family. Physiologically, ANGPT1 binds to TEK to mediate embryonic vascular development and angiogenesis [37, 38]. A previous study showed that normal lung tissues expressed constitutively high and correlated levels of ANGPT1 and TEK, which were significantly reduced in non-small cell lung cancers (NSCLC) [39]. These previous findings indicated a specified role of the ANGPT1/TEK pathway in the maintenance of the complex vasculature in normal lungs. Evidence has shown that lung cancer patients with a higher level of ANGPT1 had better survival, indicating that ANGPT1 is a prognostic marker for lung cancer, especially for predicting postoperative survival and recurrence [40]. Furthermore, a recent study demonstrated that ANGPT1 could be a potential tumor suppressor gene for lung cancer [41]. Alterations in the intron region of ANGPT1 were found in lung cancer and affected the expression level of ANGPT1, which lead to the neoplastic progression of lung cancer. Moreover, survival analysis found that high expression of ANGPT1 associated with a higher survival probability individually [41].

Although there have been no reports of P2RY1 and CALCRL involved in lung diseases and cancer, they all have been demonstrated to play important roles in other cancers, such as bladder cancer [42], prostate cancer [43], and acute myeloid leukemia [44]. They are prognostic markers or key molecules for tumorigenesis in these cancers. Therefore, they may also be potential markers for LAC and require further study. In short, our study provides some useful information and clues for future studies in LAC.

## Conclusions

Our bioinformatic analysis identified six downregulated DEGs (EDNRB, RXFP1, P2RY1, CALCRL, TEK, and ANGPT1) between lung adenocarcinoma and normal lung tissues based on two different microarray datasets. These six genes were identified as excellent prognostic markers and potential tumor suppressors, playing key roles in the initiation and progression of LAC. However, more studies are required to verify the prediction and underlying mechanisms in the near future. These data may provide novel perspectives and clues into the study of potential molecular markers and therapeutic targets in LAC.

## Supplementary Information

**Additional file 1: Table S1.** General information of the differentially expressed genes in GSE27262 datasets. **Table S1–1.** Down-regulated genes in GSE27262 datasets. **Table S1–2.** Up-regulated genes in GSE27262 datasets.

**Additional file 2: Table S2.** General information of the differentially expressed genes in GSE118370 datasets. **Table S2–1.** Down-regulated genes in GSE118370 datasets. **Table S2–2.** Up-regulated genes in GSE118370 datasets.

**Additional file 3: Table S3.** Key properties of PPI network (related to Fig. 2). **Table S3–1.** Key properties of PPI network of common DEGs (related to Fig. 2a). **Table S3–2.** Key properties of PPI network of 17 central DEGs (related to Fig. 2b).

**Additional file 4: Table S4.** Validation of the 189 common DEGs in E-GEOD-30219 datasets. **Table S4–1.** Validation of the 162 common down-regulated DEGs in E-GEOD-30219 datasets. **Table S4–2.** Validation of the 27 common up-regulated DEGs in E-GEOD-30219 datasets.

**Additional file 5: Table S5.** Validation of the 189 common DEGs in E-GEOD-19188 datasets. **Table S5–1.** Validation of the 162 common down-regulated DEGs in E-GEOD-19188 datasets. **Table S5–2.** Validation of the 27 common up-regulated DEGs in E-GEOD-19188 datasets.

## Data Availability

Dataset supporting our findings is available, at the following website: www.ncbi.nlm.nih.gov/geo/, www.ebi.ac.uk/arrayexpress/. All data generated or analyzed during this study are available from the corresponding author on reasonable request.

## Abbreviations

LAC: Lung adenocarcinoma
GEO: Gene Expression Omnibus
DEGs: Differentially expressed genes
EDNRB: Endothelin receptor type B
RXFP1: Relaxin family peptide receptor-1
P2RY1: Purinergic receptor P2Y1
CALCRL: Calcitonin receptor like receptor
TEK: TEK receptor tyrosine kinase
ANGPT1: Angiopoietin-1
PPI: Protein-protein interaction
DAVID: The Database for Annotation, Visualization and Integrated Discovery
GO: Gene ontology
KEGG pathways: Kyoto Encyclopedia of Gene and Genome pathways
STRING: Search Tool for the Retrieval of Interacting Genes
MCODE: Molecular Complex Detection
GEPIA: Gene Expression Profiling Interactive Analysis
TCGA: The Cancer Genome Atlas
